# 5-Aza Exposure Improves Reprogramming Process Through Embryoid Body Formation in Human Gingival Stem Cells

**DOI:** 10.3389/fgene.2018.00419

**Published:** 2018-10-08

**Authors:** Francesca Diomede, Nicoletta Zini, Jacopo Pizzicannella, Ilaria Merciaro, Giuseppe Pizzicannella, Monica D’Orazio, Adriano Piattelli, Oriana Trubiani

**Affiliations:** ^1^Department of Medical, Oral and Biotechnological Sciences, D’Annunzio University of Chieti-Pescara, Chieti, Italy; ^2^CNR-National Research Council of Italy, IGM, Bologna, Italy; ^3^IRCCS, Rizzoli Orthopaedic Institute, Bologna, Italy; ^4^ASL 2 Lanciano Vasto Chieti, Chieti, Italy; ^5^Division of Rheumatology and Immunology, Department of Internal Medicine, Medical University of Graz, Graz, Austria; ^6^Chair of Biomaterials Engineering, Catholic University of San Antonio of Murcia (UCAM), Murcia, Spain

**Keywords:** embryoid bodies, 5-Aza-2′-deoxycytidine, human gingival mesenchymal stem cells, DNMT1, p21

## Abstract

Embryoid bodies (EBs) are three-dimensional aggregates formed by pluripotent stem cells, including embryonic stem cells and induced pluripotent stem cells. They are used as an *in vitro* model to evaluate early extraembryonic tissue formation and differentiation process. In the adult organisms, cell differentiation is controlled and realized through the epigenetic regulation of gene expression, which consists of various mechanisms including DNA methylation. One demethylating agent is represented by 5-Azacytidine (5-Aza), considered able to induce epigenetic changes through gene derepression. Human gingival mesenchymal stem cells (hGMSCs), an easily accessible stem cells population, migrated from neural crest. They are particularly apt as an *in vitro* study model in regenerative medicine and in systemic diseases. The ability of 5-Aza treatment to induce hGMSCs toward a dedifferentiation stage and in particular versus EBs formation was investigated. For this purpose hGMSCs were treated for 48 h with 5-Aza (5 μM). After treatment, hGMSCs are organized as round 3D structures (EBs-hGMSCs). At light and transmission electron microscopy, the cells at the periphery of EBs-hGMSCs appear elongated, while ribbon-shaped cells and smaller cells with irregular shape surrounded by extracellular matrix were present in the center. By RT-PCR, EBs-hGMSCs expressed specific transcription markers related to the three germ layers as MAP-2, PAX-6 (ectoderm), MSX-1, Flk-1 (mesoderm), GATA-4, and GATA-6 (endoderm). Moreover, in EB-hGMSCs the overexpression of DNMT1 and ACH3 other than the down regulation of p21 was detectable. Immunofluorescence staining also showed a positivity for specific etodermal and mesodermal markers. In conclusion, 5-Aza was able to induce the direct conversion of adult hGMSCs into cells of three embryonic lineages: endoderm, ectoderm, and mesoderm, suggesting their possible application in autologous cell therapy for clinical organ repair.

## Introduction

Mesenchymal stem cells (MSCs) are adult and multipotent stem cells described by Friedenstein et al. (1970) as plastic-adherent cells with fibroblast-like morphology, possessing self-renewal and tissue regeneration abilities (Huang et al., 2009). Human MSCs are isolated from the bone marrow and defined as a not hematopoietic stem cell population (Chen et al., 2017).

Actually, to understand the molecular and cellular signals, that occur during the gastrulation and then in the development of the three embryonic germ layers (endoderm, mesoderm, and ectoderm), many researchers are interested in the evolving of new *in vitro* techniques simulating the *in vivo* development. In particular, the formation of embryoid bodies (EBs) *in vitro* represents a way to test the pluripotency of human stem cells and their differentiation process modulation (Papapetrou et al., 2009). EB is a spherical structure, which in itself contains cell aggregates promoting multicellular interactions, which consist of ectodermal, mesodermal, and endodermal tissues leading to cell differentiation during embryogenesis (Taru Sharma et al., 2012).

Pluripotency, self-renewal and aging are stem cell features that can be affected by epigenetic modifications. One of the most important mechanisms that regulates epigenetic modification of the genome is DNA methylation (Selvaraj et al., 2010). 5-Azacytidine (5-Aza) is a DNA demethylating agent, in particular, it plays a role as the inhibitor of DNA methyltransferase (DNMT) and it is involved in cell growth and differentiation (Abbey and Seshagiri, 2013). DNA methylation is an important mechanism involved in maintaining pluripotency and self-renewal of stem cells. 5-Aza can be used to induce stem cell differentiation in MSCs from bone marrow and adipose tissue but its role on stem cells remains contradictory (Rangappa et al., 2004; Xu et al., 2004). In the last years, various human stem cells have been described from oral cavity tissues. Oral stem cells have been isolated and characterized from exfoliated deciduous teeth (SHEDs) (Miura et al., 2003), periodontal ligament (PDLSCs) (Trubiani et al., 2016), dental follicle progenitor cells (DFPCs) (Morsczeck et al., 2005), apical papilla (SCAPs) (Sonoyama et al., 2008), dental pulp (DPSCs) (Ashri et al., 2015), and gingival tissue [Human gingival mesenchymal stem cells (hGMSCs)] (Diomede et al., 2018).

Stem cells derived from human gingiva have been previously characterized as a subpopulation of MSCs migrated from neural crest cells during tooth development with spindle-shaped appearance and rapid expansion (Rajan et al., 2016a). Moreover, the high proliferation rate, the expression of specific markers of pluripotency, the ability to differentiate into cells of all the three embryonic germ layers (Ballerini et al., 2017; Diomede et al., 2017b) as well as the ability to undergo toward neuronal differentiation make these cells apt for biological studies (Giacoppo et al., 2017).

The aim of this study was to investigate whether it was possible to achieve the direct conversion of oral adult stem cells into cells of three embryonic lineages by exposing them to a demethylating agent immediately followed by standard culture conditions.

## Materials and Methods

### Establishment and Expansion of hGMSCs

This study was approved by the d’Annunzio University Human Research Ethics Committee (No. 1711/13). All patients have been signed the informed consent as requested by rules of the Department of Medical, Oral and Biotechnological Sciences (ISO 9001:2008, RINA certified 32031/15/S). hGMSCs were isolated as previously described by Diomede et al. (2014). Gingival connective tissues were obtained during surgical treatment. Samples were washed several times, cut into small pieces, and placed at 37°C to obtain adherent hGMSCs culture. Primary hGMSCs cultures were established and maintained in MSCs growth medium chemically defined (MSCGM-CD, Lonza, Basel, Switzerland) without animal sera addiction. Cells were maintained in a humidified atmosphere 5% CO_2_ at 37°C up to 80% of confluence and detached using Trypsin/EDTA solution (TripleSelect, Life Tech, Milan, Italy). Cells at second passage were used for experiments.

### Flow Cytometric Analysis

Flow cytometric analysis was used to evaluate the immunophenotype of hGMSCs as previously reported (Rajan et al., 2016b). Cells at second passage were incubated with primary monoclonal antibodies Oct 3/4, Sox-2, SSEA-4 (Becton Dickinson, BD, San Jose, CA, United States), CD 29, CD 44, CD 73, CD 90, CD 105 (Ancell, MN, United States), CD 14, CD 34, and CD 45 (Beckman Coulter, Fullerton, CA, United States) and isotype-matched controls. Cell suspensions were then incubated with a secondary detection reagent, goat anti-mouse immunoglobulin G-phycoerythrin. Cells were acquired with a flow cytometer (FACS Calibur; BD) (Cianci et al., 2016). Data were analyzed by the FlowJo software v8.8.6 (TreeStar, Ashland, OR, United States).

### hGMSCs Differentiation

Human gingival mesenchymal stem cells at second passage were induced to osteogenic, adipogenic, and chondrogenic differentiation as previously reported by Rajan et al. (2017a). Alizarin red S staining was assessed to evaluate osteogenic differentiation, while to highlight adipogenic commitment the cells were stained with Adipo Oil Red solution. Chondrogenic differentiation was evidenced by means of Alcian blue staining. The images were captured using inverted light microscopy, Leica DMIL (Leica Microsystem, Milan, Italy) (Manescu et al., 2016).

For neurogenic differentiation hGMSCs were plated in 24-well plates and were induced for 10 days by Neurobasal-A Medium (Gibco^®^) containing B27 (2%), L-glutamine (2 mM), penicillin (100 U/ml), streptomycin (100 mg/ml), amphotericin B (5 mg/ml) (neuroinductive medium), and supplemented with basic Fibroblast Growth Factor (bFGF, 20 ng/ml) (TemaRicerca, Milan, Italy) as previously reported (Trubiani et al., 2016). To evaluate the differentiation, cells were processed for βIII-tubulin immunostaining detection and were observed under confocal laser scanning microscope, LSM510META (Zeiss, Jena, Germany).

### Treatment With 5-Azacytidine (5-Aza)

Human gingival mesenchymal stem cells were treated with 5-Aza (5 μm) for 48 h in a humidified atmosphere 5% CO_2_ at 37°C. Subsequently, 5-Aza treatment was removed and then cells were cultured in MSCGM-CD (Lonza) for 10 days. Untreated cells were used as control.

### Morphological Evaluation of EBs-hGMSCs

#### Inverted Light Microscopy

After 48 h of 5-Aza treatment, the morphology of the EBs-hGMSCs were observed under the inverted light microscope (Leica DMIL, Leica Microsystem) at 0, 5, 10, and 48 h.

#### Light and Transmission Electron Microscopy

Embryoid bodies-hGMSCs were fixed with 2.5% glutaraldehyde in 0.1 M cacodylate buffer pH 7.4 for 1 h at room temperature (RT). After fixation, the samples were post-fixed with 1% osmium tetroxide, dehydrated in a graded series of ethanol, and embedded in Epon. Semithin sections were stained with toluidine blue and used for light microscopy analysis. The sections were observed with a Zeiss Axiophot apparatus, and images were captured using a Nikon digital camera Digital Sight (Diomede et al., 2016b). Thin sections were stained with uranyl acetate and lead citrate; some sections were also stained with tannic acid, and finally observed with a Zeiss EM 109 transmission electron microscope. Images were captured using a Nikon digital camera Dmx 1200F and ACT-1 software.

#### RT-PCR Assay

Total RNA was extracted from EBs-hGMSCs and hGMSCs using the RNeasy Mini Kit (Quiagen, Hilden, Germany). 2 μg of RNA from each sample was reverse transcribed using the High Capacity RNA-tocDNA Kit (Applied Biosystems, Foster, United Kingdom). To analyze osteogenic, adipogenic, chondrogenic, and neurogenic differentiation, appropriate mRNA transcripts were evaluated. In particular, RUNX-2 and ALP for osteogenesis (Diomede et al., 2016a), PPARγ and FABP4 for adipogenesis (Rajan et al., 2017a), ACAN and COL2A1 for chondrogenesis (Rajan et al., 2017a), and Nestin and Enolase for neurogenesis (Diomede et al., 2017a) were analyzed.

To verify the differentiation potential of EBs-hGMSCs *in vitro*, expression analysis of molecular markers as paired box-6 (PAX-6) and microtubule-associated protein 2 (MAP2), fetal liver kinase-1 (FLK-1) and homeobox protein (MSX-1), GATA-binding factor 4 and 6 (GATA-4, GATA-6), for ectoderm, mesoderm, and endoderm, respectively, were carried out. Total RNA from EBs-hGMSCs and hGMSCs was purified with RNA Purification Kit (Norgen Biotek Corp., Ontario, CA, United States) according to the manufacturer’s instructions. Real-Time PCR (RT-PCR) was carried out with the Mastercycler ep realplex RT-PCR system (Eppendorf, Hamburg, Germany). Commercially available TaqMan Gene Expression Assays (PAX-6 Hs01088114_m1; MAP-2 Hs00258900_m1; Flk-1 Hs00911700_m1; MSX-1 Hs00427183_m1; GATA-4 Hs00171403_m1; and GATA-6 Hs00232018_m1) and the TaqMan Universal PCR Master Mix (Applied Biosystems, Foster City, CA, United States) were used according to standard protocols. Beta-2 microglobulin (B2M Hs99999907_m1) (Applied Biosystems, Foster City, CA, United States) was used as template normalization. RT-PCR was performed in three independent experiments, duplicate determinations were carried out for each sample.

#### Immunofluorescence Staining and Confocal Laser Scanning Microscopy

Embryoid bodies-hGMSCs were fixed in 4% paraformaldehyde (PFA) (BioOptica, Milan, Italy) for 1 h at RT. Then samples were permeabilized with 1% Triton X-100 (BioOptica) for 5 min and treated with blocking buffer made of 2% BSA in PBS for 1 h. The following primary monoclonal antibodies were used: anti human PAX-6 (1:50; Abcam, Cambridge, United Kingdom) and anti-Brachyury (1:250; Abcam). Then, cells were incubated for 1 h at 37°C with Alexa Fluor 568 and Alexa Fluor 488, red and green fluorescence, respectively, conjugated goat anti-rabbit secondary antibodies (1:200) (Molecular Probes). Glass coverslips were placed upside down on glass slides and mounted with Prolong antifade (Molecular Probes). Samples were observed with Zeiss LSM510META confocal system (Zeiss, Jena, Germany) connected to an inverted Zeiss Axiovert 200 microscope equipped with a Plan Neofluar oil-immersion objectives (Diomede et al., 2017c). Images were collected using an argon laser beam with excitation lines at 488 nm and a helium-neon source at 543 and 665 nm.

#### Western Blot

Forty micrograms of proteins obtained from EBs-hGMSCs and hGMSCs were separated on SDS-PAGE and subsequently transferred to PVDF membrane using a SEMI-dry blotting apparatus (Bio-Rad Laboratories Srl, MI, Italy). Membranes were saturated for 2 h at RT in blocking buffer (1 × PBS, 5% milk, 0.1% Tween-20), then incubated overnight at 4°C in blocking buffer containing primary antibodies: mouse anti-DNMT1 (1:200), rabbit anti-acetyl-histone H3 (1:500), mouse anti-p21 (1:500), and mouse anti-β-actin (Santa Cruz Biotechnology, Santa Cruz, CA) (Trubiani et al., 1996). After four washes in PBS containing 0.1% Tween-20, samples were incubated for 1 h at RT with peroxidase-conjugated secondary antibody anti-mouse and anti-rabbit, respectively, diluted 1:2000 in 1× PBS, 2.5% milk, 0.1% Tween (Libro et al., 2016). Bands were visualized and quantified by the ECL method with Alliance 2.7 (UVItec Limited, Cambridge, United Kingdom).

### Statistical Analysis

Gene expression and protein levels were compared by means of the Student’s *t*-test for unpaired observations and One way ANOVA for multiple comparisons. *P*-value < 0.05 was considered statistically significant. SPSS software (IBM, North Castel, NY, United States) was used to perform statistical evaluation.

## Results

### hGMSCs Immunophenotype

**FIGURE 1:**
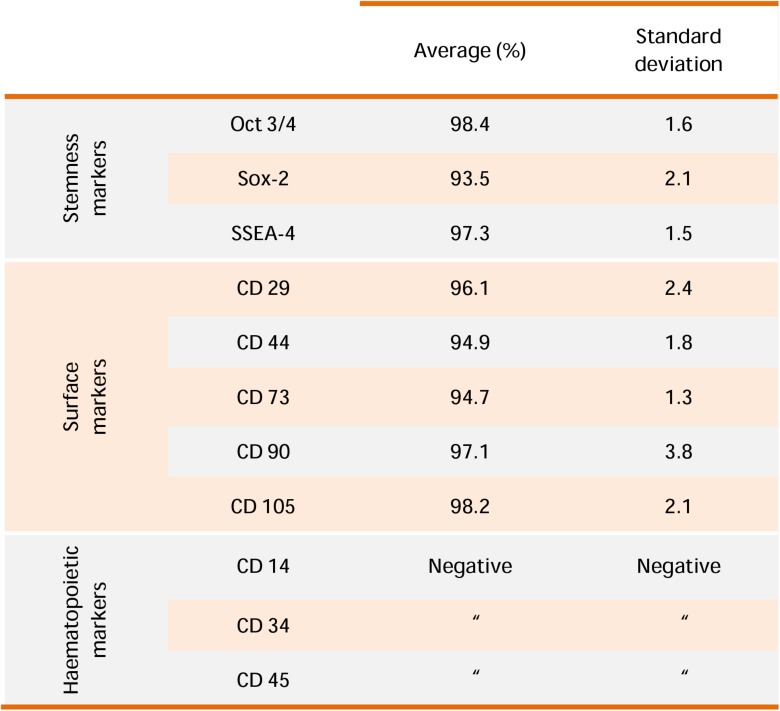
hGMSCs characterization. Representative flow cytometry of hGMSCs (*n* = 5). Cells express Oct 3/4, Sox-2, SSEA-4, CD 29, CD 44, CD 73, CD 90, and CD 105; while are negative for CD 14, CD 34, and CD 45.

Human gingival MSCs expressed high cell surface levels related to MSC markers: Oct 3/4, Sox-2, SSEA-4, CD 29, CD 44, CD 73, CD 90, and CD 105, while lacked the expression of hematopoietic markers CD 14, CD 34, and CD 45 (**Figure 1**).

### hGMSCs Differentiation Ability

**FIGURE 2:**
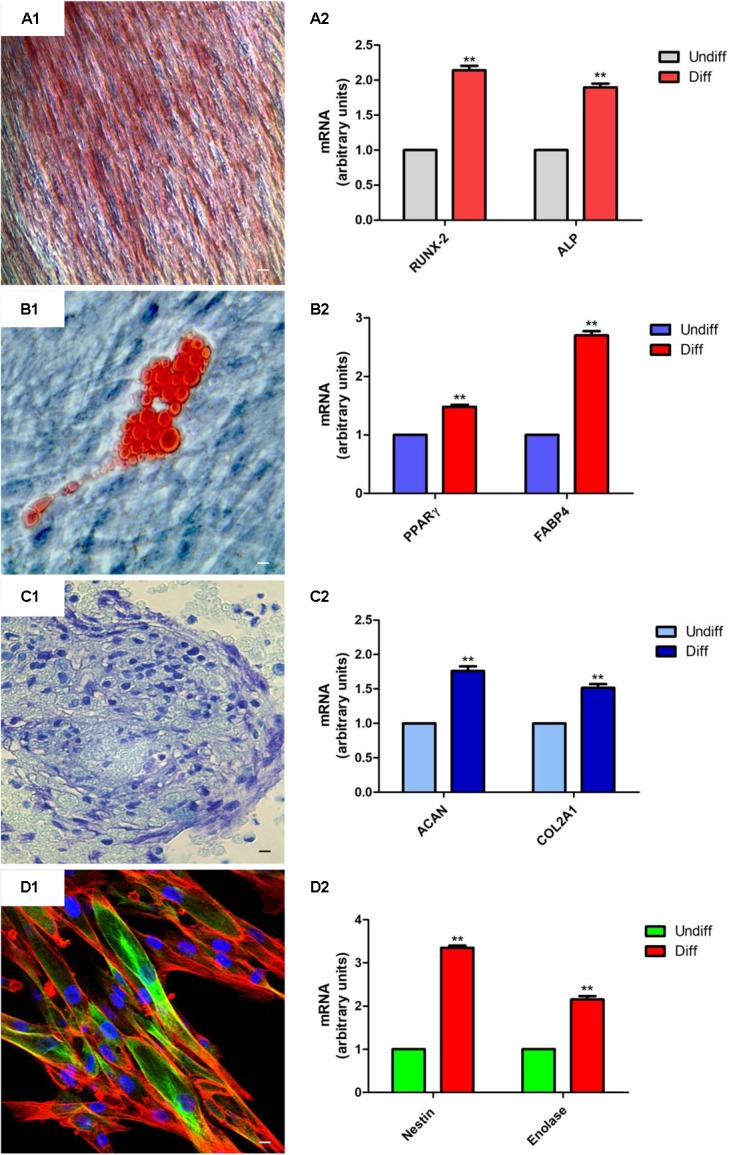
hGMSCs differentiation ability. **(A1)** Osteogenic differentiation of hGMSCs stained with Alizarin red S solution. **(A2)** RT-PCR of osteogenic related markers, RUNX2 and ALP. **(B1)** Adipogenic differentiation of hGMSCs stained with Oil Red to highlight cytoplasmic lipid droplets. **(B2)** RT-PCR of adipogenic related markers, PPARγ and FABP4. **(C1)** Chondrogenic differentiation of hGMSCs stained with Alcian blue solution. **(C2)** RT-PCR of chondrogenic related markers, ACAN and COL2A1 after 28 days of induction. **(D1)** Neurogenic differentiation was assessed by labeling with green-fluorescent marker, βIII-tubulin, cytoskeleton actin in red and nuclei in blue. **(D2)** RT-PCR of neurogenic related markers, Nestin and Enolase after 10 days of induction. Scale bar = 10 μm. ^∗∗^*p* < 0.01.

The multipotent potential of hGMSCs for differentiation in osteogenic, adipogenic, chondrogenic, and neurogenic lineages was determined by staining with Alizarin Red S, Oil Red O, Alcian blue and βIII-tubulin, respectively, after culture in specific induction media (**Figures 2A1,B1,C1,D1**). To confirm the obtained data, RT-PCR was assessed for all differentiated samples. RUNX2 and ALP were overexpressed in osteogenic committed cells (**Figure 2A2**), PPARγ and FABP4 were upregulated in adipogenic differentiated cells (**Figure 2B2**), ACAN and COL2A1 were upregulated in cells cultured in chondrogenic medium (**Figure 2C2**). Moreover, cells maintained under neurogenic conditions showed a high expression of neurogenic related markers, nestin and enolase, when compared with the undifferentiated cells (**Figure 2D2**).

### EBs Morphological Analyses

After 48 h of 5-Aza treatment the medium was replaced with fresh MSCGM-CD and hGMSCs were observed under inverted light microscopy at following timepoints: 0, 5, 10, and 48 h.

**FIGURE 3:**
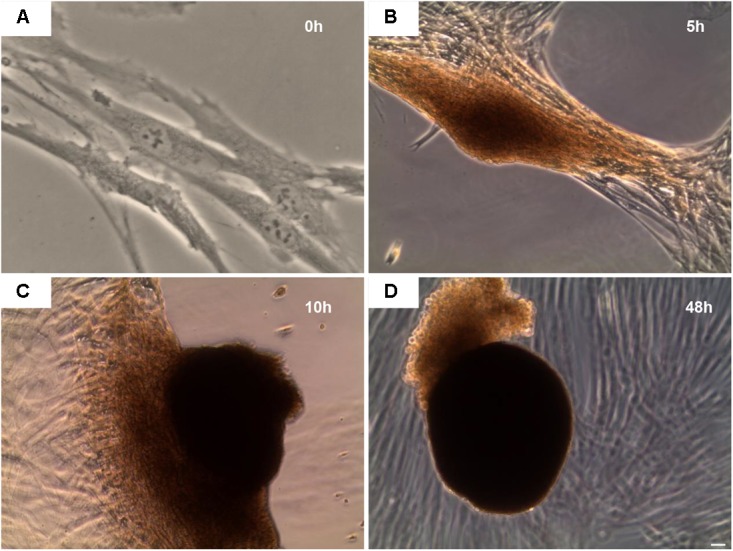
Inverted light microscope evaluation. hGMSCs treated with 5-Aza develop embryoid bodies like structure. Observations were carried out at different endpoint: **(A)** 0 h, **(B)** 5 h, **(C)** 10 h, and **(D)** 48 h. Mag: 10×.

At 0 h, hGMSCs were plastic-adherent and showed a fibroblast like morphology (**Figure 3A**). Starting to 5 h till 48 h cells changed their morphological arrangement, in fact, adherent-cells to the plastic substrate formed a small cellular aggregate (EBs-hGMSCs) completely detached from the bottom well (**Figures 3B–D**).

**FIGURE 4:**
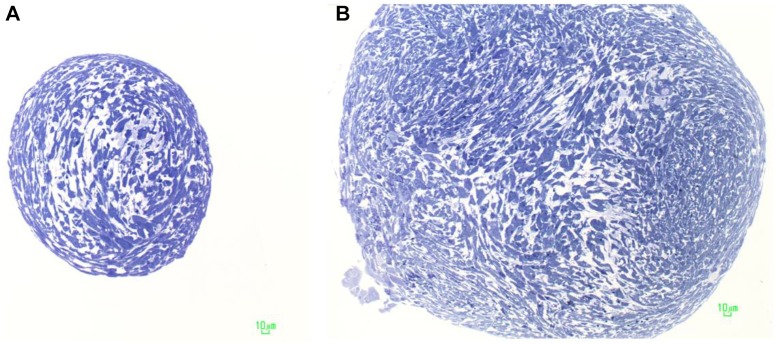
Characterization of embryoid bodies. Light microscopy of an EB-hGMSCs. Semithin sections stained with toluidine blue. **(A)** Section at the top of the samples; and **(B)** section in the middle of the EB.

Light microscopy of toluidine blue-stained semithin sections of resin-embedded samples after 10 days of culture and after 5-Aza treatment revealed EBs-hGMSCs as spherical dense cellular bodies (**Figure 4**).

**FIGURE 5:**
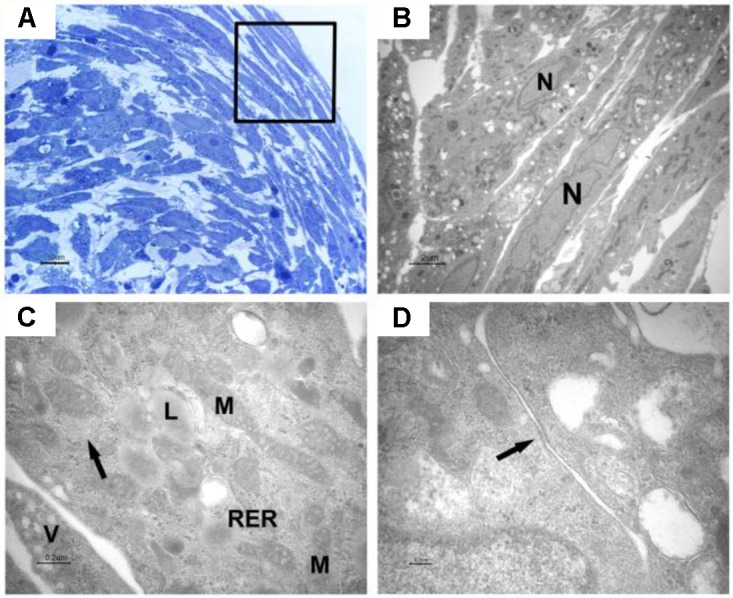
Light and transmission electron microscopy of EB-hGMSCs periphery. **(A)** Light microscopy of semithin section stained with toluidine blue at high magnification. The square marked peripheric area where the cells are elongated. Bar: 10 μm. **(B–D)** Transmission electron microscopy. **(B)** A magnification of an area similar to that marked by square in **(A)** shows some layers of elongated cells with euchromatic nuclei (N). Bar: 2 μm. **(C)** At higher magnification, filaments (arrow), lipid droplets (L), mitochondria (M), rough endoplasmic reticulum (RER), and vesicles (V) are visible in the cytoplasm. Bar: 0.2 μm. **(D)** Plasma membrane thickening is present (arrow). Bar: 0.1 μm.

**FIGURE 6:**
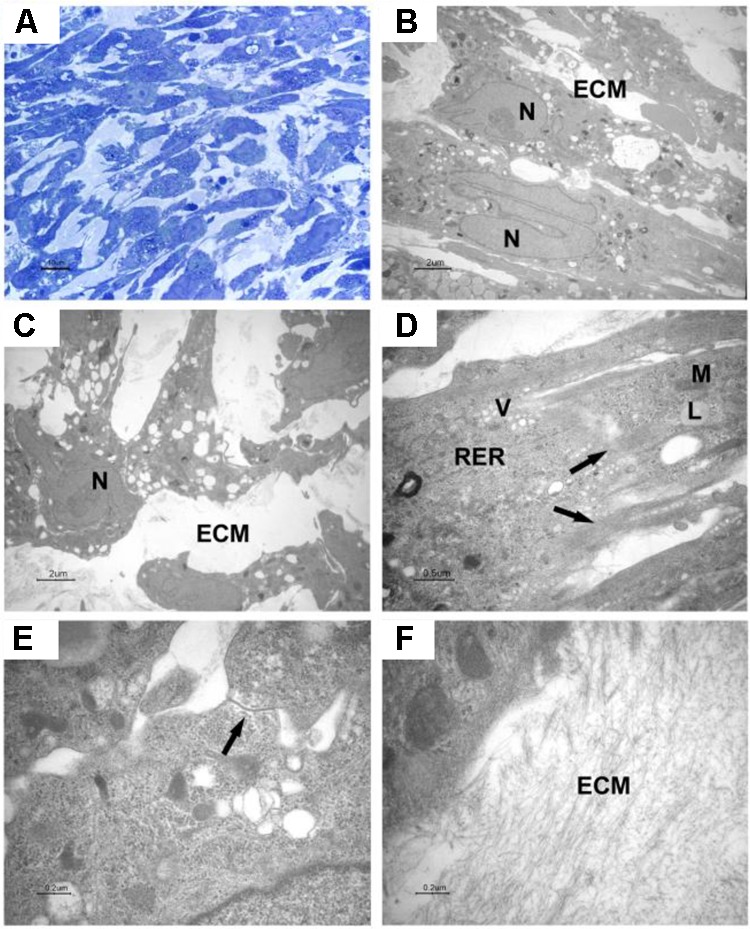
Light and transmission electron microscopy of EB-hGMSCs centre. **(A)** Light microscopy of semithin section stained with toluidine blue at high magnification. Bar: 10 μm. **(B–F)** Transmission electron microscopy. **(B,C)** Magnifications of areas similar to that shown in **(A)** shows ellipsoid **(B)** and irregular contour cells **(C)** with euchromatic nuclei (N), the cells are immersed in extracellular matrix (ECM). Bars: 2 μm. **(D)** At higher magnification, filaments (arrows), lipid droplets (L), mitochondria (M), vesicles (V), and rough endoplasmic reticulum (RER) are visible in the cytoplasm. Bar: 0.5 μm. **(E)** A junctional complex is present between two cells (arrow). Bar: 0.2 μm. **(F)** Higher magnification of extracellular matrix (ECM). Bar: 0.2 μm.

The cells were elongated at the periphery of EBs-hGMSCs as shown by light microscopy image at higher magnification (black square in **Figure 5A**) and confirmed by transmission electron microscopy analysis (**Figure 5B**). The cells showed euchromatic nuclei and, at higher magnification, filaments, mitochondria, lipid droplets, rough endoplasmic reticulum, and vesicles were visible in the cytoplasm (**Figure 5C**). Plasma membrane thickenings were also observed (**Figure 5D**). Light microscopy observation at higher magnification of EBs-hGMSCs center, showed ribbon-shaped cells and smaller cells with irregular shape (**Figure 6A**) also highlighted by electron microscopy analysis (**Figures 6B,C**). The cells showed euchromatic nuclei. Filaments, mitochondria, lipid droplets, rough endoplasmic reticulum, and vesicles were present in the cytoplasm (**Figure 6D**). Some junctional complexes were observed to connect adjacent cells (**Figure 6E**). Ultrastructural observation showed that the cells were surrounded by extracellular matrix (**Figures 6B,C**) that appeared predominantly fibrillar at higher magnifications (**Figure 6F**).

### Gene Expression

**FIGURE 7:**
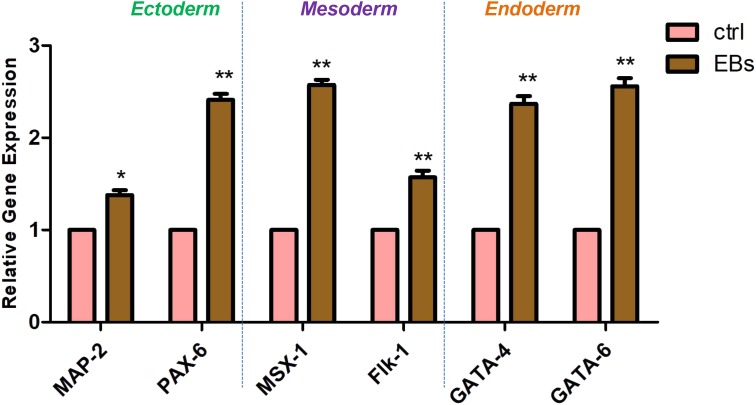
Gene expression. Analysis of lineage-specific markers for ectoderm (MAP-2 and PAX-6), mesoderm (MSX-1 and Flk-1), and endoderm (GATA-4 and GATA-6) layers in hGMSCs (ctrl) and in EBs-hGMSCs (EBs). ^∗∗^*p* < 0.01 and ^∗^*p* < 0.05.

Cell fate decision of pluripotent embryonic stem cells is dictated by the activation and repression of specific sets of lineage-specific genes. To determine the EBs-hGMSCs functionality quantitative RT-PCR (qRT-PCR) assays for lineage-specific genes was performed. EBs-hGMSCs showed a significantly increased expression of ectoderm (MAP-2 and PAX-6), mesoderm (MSX-1 and Flk-1), and endoderm (GATA-4 and GATA-6) specific markers compared to untreated hGMSCs (**Figure 7**).

### Immunofluorescence Staining

**FIGURE 8:**
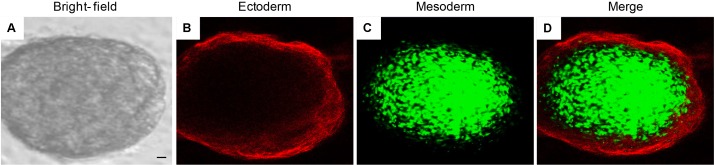
Immunofluorescence staining of EB-hGMSCs. Images of within the same colony. Representative pictures of ectoderm and mesoderm germ layers of EB-hGMSCs. **(A)** Bright field acquired image, **(B)** PAX6 ectoderm marker staining (red fluorescence), **(C)** Brachyury mesoderm marker staining (green fluorescence), and **(D)** merge image of above mentioned two channels. Scale bar: 50 μm. Results are representatives of three different experimental replicates.

Pictures at bright field of EBs-hGMSCs showed a spherical morphology (**Figure 8A**). The immunostaining of the same sample showed a positivity of PAX6 (ectoderm marker) localized at peripheral level (**Figure 8B**), while Brachyury (mesoderm marker) positive cells were localized into a middle area (**Figure 8C**), demonstrating the specific germ layer 3D-organization (**Figure 8D**).

### Western Blot Analysis

**FIGURE 9:**
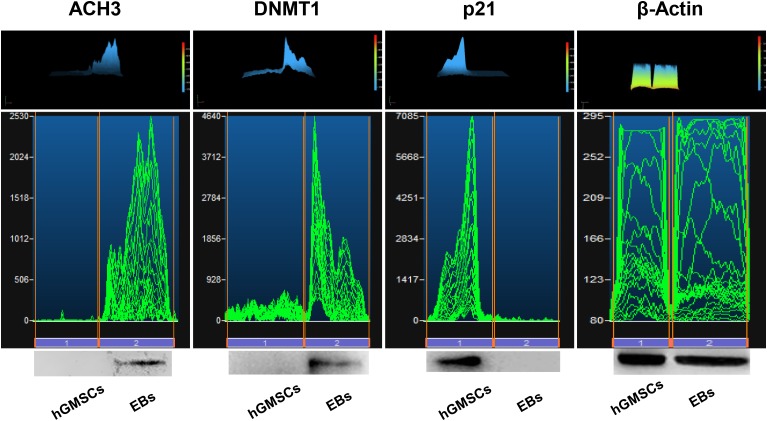
Protein levels. Western blot analysis showed an over expression of ACH3 and DNMT1 and a down expression of p21 in EBs-hGMSCs (EBs) when compared to the untreated hGMSCs. Beta-actin has been used as housekeeping protein.

Protein levels were measured using western blotting analysis. ACH3 and DNMT1 were overexpressed in EBs-hGMSCs meanwhile p21 was downregulated when compared to untreated hGMSCs (**Figure 9**).

## Discussion

Vertebrate organ development is related to inductive interactions between the epithelium and the adjacent mesenchyme (Slavkin, 1984; Yasugi, 1993). During the development, the loss of pluripotency in blastomeres is accompanied by extensive DNA methylation changes, resulting in a differentiation process in embryos.

The exposure to 5-Aza, a DNA demethylating agent, inhibits DNA methyltransferase activity and re-establishes the expression of pluripotency markers in differentiated mouse ESCs (Santos and Dean, 2004). In fact, mouse trophoblast cells and ESCs cultured with 5-Aza (1 μM) expressed OCT4 and NANOG embryonic markers (Hattori et al., 2004), and were able to differentiate *in vivo* (Lim et al., 2011).

*In vivo*, dental epithelium originates from the oral ectoderm during tooth germ development. The dental mesenchyme is derived from neural crest cells that take origin from the ectoderm (Thesleff and Hurmerinta, 1981; Balic and Thesleff, 2015). Starting from these considerations, cells derived from oral cavity tissue could represent a platform to analyze the multiple processes linked to precocious embryogenic stages.

Actually, a common method for producing different cell lineages for numerous applications in cell biology and in regenerative medicine is represented by EBs obtained from ESCs.

In this study, a new experimental model to obtain de-differentiated cells able to develop EBs starting from hGMSCs exposed to 5-Aza for 48 h was used.

Recently, it has been demonstrated that hGMSCs, highly proliferative until long term passage, were relatively uniform in terms of ultrastructure and expression of surface antigen markers linked with mesenchymal profile, such as CD 13, CD 29, CD 44, CD 73, CD 90, CD 105, CD 166, and HLA-ABC (Diomede et al., 2017b; Rajan et al., 2017b). The expression of selective embryonic markers OCT4, SSEA-4, SOX2, and NANOG were also detected until passage 15 (P15) (Diomede et al., 2017b) and in addition, the heterogeneous expression of other embryonic markers such as CDYL, FOXC1, HESX1, JARID2, MEIS1, and MYST3 at P15 in hGMSCs has been observed (Diomede et al., 2017b).

Human gingival mesenchymal stem cells are spontaneously able to start a differentiation process toward neural progenitor cells after extended passaging. In fact, the morphology of hGMSCs was changed from typical fibroblast-like shape to sphere shaped cells, observable at passage 41 (Rajan et al., 2017b). Next generation transcriptomics sequencing displayed increased expression of neurogenesis-associated genes, including NRXN2, EFNA5, GDAP1L1, LHX9, and HDAC2. In addition, *de novo* expression of neural precursor genes, such as NRN1, PHOX2B, VANGL2, and NTRK3 were detected at passage 41 (Rajan et al., 2017b).

Light and electron microscope demonstrated that 5-Aza induced *in vitro* hGMSCs aggregation in round cluster of different size, typical characteristic of EBs formation. Transmission electron microscope observations confirmed that EBs generated from hGMSCs (EBs-hGMSCs) showed a structural organization with formation of junctional complexes. Moreover, the role of 5-Aza, on hGMSCs culture, was also related to epigenetic regulatory mechanisms and associated in gene expression changes. In fact, 5-Aza treatment was associated in EBs-hGMSCs with DNMT1 upregulation and with histone acetylation improvement.

EBs represents the early stage of lineage-specific differentiation toward many lineages such as cardiac, neural and hematopoietic (Dang et al., 2002; Sargent et al., 2009; Sathananthan, 2011). Although EBs permit the generation of cells to all three primary germ layers, the differentiation outcomes are highly dependent upon the quality of EBs, which is affected by the medium conditions, the cell numbers, and the sizes of EBs (Khoo et al., 2005).

In the present experimental model, RT-PCR performed on EBs-hGMSCs, showed a positive expression for MAP-2, PAX-6, Flk-1, MSX-1, GATA-4, and GATA-6 genes, representative of all three germ layers.

N-CAM is an integral membrane glycoprotein that is present on the surfaces of most peripheral and central neurons. This molecule is thought to mediate a variety of intercellular adhesive interactions in the nervous system (Bock, 1998).

MAP2K2 is an element of a signaling called the RAS/MAPK pathway, which transmits chemical signals from outside the cell to the cell’s nucleus. RAS/MAPK signaling helps control of growth and proliferation, differentiation, cell movement, and apoptosis (Duesbery and Vande Woude, 2006; Scholl et al., 2007).

MSX-1 gene is involved in the protein assembly regulating the activity of other genes. Moreover, it is considered as part of a larger family of homeobox genes acting during early development to control the formation of many body structures.

The GATA family of transcription factors consists of six proteins (GATA1-6) involved in a variety of physiological and pathological processes. GATA1/2/3 are required for differentiation of mesoderm and ectoderm-derived tissues, while GATA4/5/6 are implicated in development and differentiation of endoderm- and mesoderm-derived tissues, such as induction of ESC differentiation, cardiovascular embryogenesis and guidance of epithelial cell differentiation in the adult (Lentjes et al., 2016). Ectodermal PAX-6 transcription factor, detected during embryonic development and involved in eye and brain development, was upregulated in EBs (Yan et al., 2010).

Remarkably, immunostaining of PAX-6 and Brachyury of EBs-hGMSCs observed under confocal microscopy, put in evidence the specific spatial organization in germ layer, as ectodermic and mesodermic layers, respectively (Poh et al., 2014).

In conclusion, this study demonstrates a simple and efficient method to develop EBs from oral cavity derived stem cells, according to the study showing that DPSCs are able to form embryoid-body-like structures *in vitro* containing cells derived from all three embryonic germ layers when injected in nude mice (Yan et al., 2010). Moreover, it has been demonstrated that oral derived stem cells possess higher reprogramming efficiency than dermal fibroblasts, thus prove that these cells are more amenable for reprogramming (Pisal et al., 2018) and that transgene-free-DSC iPSCs are capable to undergo toward neurogenic differentiation till to functional neurons stage *in vitro* (El Ayachi et al., 2018).

The study outcomes were focused on the potential role of 5-Aza and provide a potential link between epigenetic modification and cell differentiation.

Epigenetic modification, as DNA methylation, histone modification, and chromatin remodeling, are crucial for cellular differentiation process, development, and gene expression (Yun et al., 2012). Histone modifiers, such as histone acetyltransferases (HAT) and histone deacetylases (HDAC), are able to control gene transcription during development and pan-HDAC inhibitors have been shown to block the differentiation *in vitro* (Haberland et al., 2009; Yu et al., 2010). In this culture system ACH3 is expressed as DNMT1, conversely as aspected p21 is absent. p21 is a senescence markers that is expressed in long-term passage cell culture (Diomede et al., 2017b). Further investigations will be necessary to understand the differentiation ability of hGMSCs, their biology machinery and their possible application in autologous cell therapy for clinical organ repair.

Within the limitations of this study, it could be conceivable that 5-Aza induces a de-differentiation process related to a EBs formation in our culture system, according with our results showing the expression of the ecto-, meso-, and endo-dermal markers and the morphological changes of hGMSCs. Then, the new cell technology that use adult stem cells from an easily accessible source, as the oral tissues, could open new opportunity in stem cell research in order to provide cells able to differentiate in multiple lineages.

## Author Contributions

FD, NZ, and OT wrote the main manuscript text. FD and IM performed *in vitro* hPDLSCs experiments and data analysis. NZ performed TEM experiments and data analysis. FD, JP, and MD’O contributed to sample collection and cytofluorimetric analyses. JP, GP, and AP revised the manuscript. OT conceived and designed the experiments. All authors reviewed the manuscript.

## Conflict of Interest Statement

The authors declare that the research was conducted in the absence of any commercial or financial relationships that could be construed as a potential conflict of interest.

## References

[B1] Abbey D. Seshagiri P. B. (2013). Aza-induced cardiomyocyte differentiation of P19 EC-cells by epigenetic co-regulation and ERK signaling. *Gene* 526 364–373. 10.1016/j.gene.2013.05.044 23747406

[B2] Ashri N. Y. Ajlan S. A. Aldahmash A. M. (2015). Dental pulp stem cells. Biology and use for periodontal tissue engineering. *Saudi Med. J.* 36 1391–1399. 10.15537/smj.2015.12.12750 26620980 PMC4707394

[B3] Balic A. Thesleff I. (2015). Tissue interactions regulating tooth development and renewal. *Curr. Top. Dev. Biol.* 115 157–186. 10.1016/bs.ctdb.2015.07.006 26589925

[B4] Ballerini P. Diomede F. Petragnani N. Cicchitti S. Merciaro I. Cavalcanti M. F. X. B. (2017). Conditioned medium from relapsing-remitting multiple sclerosis patients reduces the expression and release of inflammatory cytokines induced by LPS-gingivalis in THP-1 and MO3.13 cell lines. *Cytokine* 96 261–272. 10.1016/j.cyto.2017.04.022 28511117

[B5] Bock E. (1998). Structure and function of the neural cell adhesion molecule, NCAM. *J. Neurochem.* 71 S27–S27.

[B6] Chen C. Y. Tseng K. Y. Lai Y. L. Chen Y. S. Lin F. H. Lin S. K. (2017). Overexpression of insulin-like growth Factor 1 Enhanced the Osteogenic capability of aging bone marrow mesenchymal stem cells. *Theranostics* 7 1598–1611. 10.7150/thno.16637 28529639 PMC5436515

[B7] Cianci E. Recchiuti A. Trubiani O. Diomede F. Marchisio M. Miscia S. (2016). Human periodontal stem cells release specialized proresolving mediators and carry immunomodulatory and prohealing properties regulated by lipoxins. *Stem Cells Transl. Med.* 5 20–32. 10.5966/sctm.2015-0163 26607175 PMC4704879

[B8] Dang S. M. Kyba M. Perlingeiro R. Daley G. Q. Zandstra P. W. (2002). Efficiency of embryoid body formation and hematopoietic development from embryonic stem cells in different culture systems. *Biotechnol. Bioeng.* 78 442–453. 10.1002/bit.10220 11948451

[B9] Diomede F. Caputi S. Merciaro I. Frisone S. D’arcangelo C. Piattelli A. (2014). Pro-inflammatory cytokine release and cell growth inhibition in primary human oral cells after exposure to endodontic sealer. *Int. Endod. J.* 47 864–872. 10.1111/iej.12230 24325570

[B10] Diomede F. Gugliandolo A. Scionti D. Merciaro I. Cavalcanti M. F. X. B. Mazzon E. (2018). Biotherapeutic effect of gingival stem cells conditioned medium in bone tissue restoration. *Int. J. Mol. Sci.* 19:329. 10.3390/ijms19020329 29360771 PMC5855551

[B11] Diomede F. Merciaro I. Martinotti S. Cavalcanti M. F. Caputi S. Mazzon E. (2016a). miR-2861 is involved in osteogenic commitment of human periodontal ligament stem cells grown onto 3D scaffold. *J. Biol. Regul. Homeost. Agents* 30 1009–1018. 28078846

[B12] Diomede F. Zini N. Gatta V. Fulle S. Merciaro I. D’aurora M. (2016b). Human periodontal ligament stem cells cultured onto cortico-cancellous scaffold drive bone regenerative process. *Eur. Cells Materials* 32 181–201. 27633707 10.22203/ecm.v032a12

[B13] Diomede F. Rajan T. S. D’aurora M. Bramanti P. Merciaro I. Marchisio M. (2017a). Stemness characteristics of periodontal ligament stem cells from donors and multiple sclerosis patients: a comparative study. *Stem Cells Int.* 2017:1606125. 10.1155/2017/1606125 29387088 PMC5745749

[B14] Diomede F. Rajan T. S. Gatta V. D’aurora M. Merciaro I. Marchisio M. (2017b). Stemness maintenance properties in human oral stem cells after long-term passage. *Stem Cells Int.* 2017:5651287. 10.1155/2017/5651287 28469672 PMC5392399

[B15] Diomede F. Zingariello M. Cavalcanti M. Merciaro I. Pizzicannella J. De Isla N. (2017c). MyD88/ERK/NFkB pathways and pro-inflammatory cytokines release in periodontal ligament stem cells stimulated by *Porphyromonas gingivalis*. *Eur. J. Histochem.* 61:2791. 10.4081/ejh.2017.2791 28735521 PMC5452629

[B16] Duesbery N. Vande Woude G. (2006). BRAF and MEK mutations make a late entrance. *Sci. STKE* 2006:pe15. 16569817 10.1126/stke.3282006pe15

[B17] El Ayachi I. Zhang J. Zou X. Y. Li D. Yu Z. Wei W. (2018). Human dental stem cell derived transgene-free iPSCs generate functional neurons via embryoid body-mediated and direct induction methods. *J. Tissue Eng. Regen. Med.* 12 e1836–e1851. 10.1002/term.2615 29139614 PMC6482049

[B18] Friedenstein A. J. Chailakhjan R. K. Lalykina K. S. (1970). The development of fibroblast colonies in monolayer cultures of guinea-pig bone marrow and spleen cells. *Cell Tissue Kinet.* 3 393–403. 5523063 10.1111/j.1365-2184.1970.tb00347.x

[B19] Giacoppo S. Thangavelu S. R. Diomede F. Bramanti P. Conti P. Trubiani O. (2017). Anti-inflammatory effects of hypoxia-preconditioned human periodontal ligament cell secretome in an experimental model of multiple sclerosis: a key role of IL-37. *FASEB J.* 31 5592–5608. 10.1096/fj.201700524R 28842429 PMC5690382

[B20] Haberland M. Montgomery R. L. Olson E. N. (2009). The many roles of histone deacetylases in development and physiology: implications for disease and therapy. *Nat. Rev. Genet.* 10 32–42. 10.1038/nrg2485 19065135 PMC3215088

[B21] Hattori N. Nishino K. Ko Y. G. Hattori N. Ohgane J. Tanaka S. (2004). Epigenetic control of mouse Oct-4 gene expression in embryonic stem cells and trophoblast stem cells. *J. Biol. Chem.* 279 17063–17069. 14761969 10.1074/jbc.M309002200

[B22] Huang G. T. J. Gronthos S. Shi S. (2009). Mesenchymal stem cells derived from dental tissues vs. those from other sources: their biology and role in regenerative medicine. *J. Dent. Res.* 88 792–806. 10.1177/0022034509340867 19767575 PMC2830488

[B23] Khoo M. L. M. Mcquade L. R. Smith M. S. R. Lees J. G. Sidhu K. S. Tuch B. E. (2005). Growth and differentiation of embryoid bodies derived from human embryonic stem cells: effect of glucose and basic fibroblast growth factor. *Biol. Reprod.* 73 1147–1156. 16079311 10.1095/biolreprod.104.036673

[B24] Lentjes M. H. Niessen H. E. Akiyama Y. De Bruine A. P. Melotte V. Van Engeland M. (2016). The emerging role of GATA transcription factors in development and disease. *Expert Rev. Mol. Med.* 18:e3. 10.1017/erm.2016.2 26953528 PMC4836206

[B25] Libro R. Scionti D. Diomede F. Marchisio M. Grassi G. Pollastro F. (2016). Cannabidiol modulates the immunophenotype and inhibits the activation of the inflammasome in human gingival mesenchymal stem cells. *Front. Physiol.* 7:559. 10.3389/fphys.2016.00559 27932991 PMC5121123

[B26] Lim M. L. Vassiliev I. Richings N. M. Firsova A. B. Zhang C. Verma P. J. (2011). A novel, efficient method to derive bovine and mouse embryonic stem cells with in vivo differentiation potential by treatment with 5-azacytidine. *Theriogenology* 76 133–142. 10.1016/j.theriogenology.2011.01.027 21396694

[B27] Manescu A. Giuliani A. Mohammadi S. Tromba G. Mazzoni S. Diomede F. (2016). Osteogenic potential of dualblocks cultured with human periodontal ligament stem cells: in vitro and synchrotron microtomography study. *J. Periodontal Res.* 51 112–124. 10.1111/jre.12289 26094874

[B28] Miura M. Gronthos S. Zhao M. R. Lu B. Fisher L. W. Robey P. G. (2003). SHED: stem cells from human exfoliated deciduous teeth. *Proc. Natl. Acad. Sci. U.S.A.* 100 5807–5812. 10.1073/pnas.0937635100 12716973 PMC156282

[B29] Morsczeck C. Gotz W. Schierholz J. Zellhofer F. Kuhn U. Mohl C. (2005). Isolation of precursor cells (PCs) from human dental follicle of wisdom teeth. *Matrix Biol.* 24 155–165. 10.1016/j.matbio.2004.12.004 15890265

[B30] Papapetrou E. P. Tomishima M. J. Chambers S. M. Mica Y. Reed E. Menon J. (2009). Stoichiometric and temporal requirements of Oct4, Sox2, Klf4, and c-Myc expression for efficient human iPSC induction and differentiation. *Proc. Natl. Acad. Sci. U.S.A.* 106 12759–12764. 10.1073/pnas.0904825106 19549847 PMC2722286

[B31] Pisal R. V. Suchanek J. Siller R. Soukup T. Hrebikova H. Bezrouk A. (2018). Directed reprogramming of comprehensively characterized dental pulp stem cells extracted from natal tooth. *Sci. Rep.* 8:6168. 10.1038/s41598-018-24421-z 29670257 PMC5906561

[B32] Poh Y. C. Chen J. Hong Y. Yi H. Zhang S. Chen J. (2014). Generation of organized germ layers from a single mouse embryonic stem cell. *Nat. Commun.* 5:4000. 10.1038/ncomms5000 24873804 PMC4050279

[B33] Rajan T. S. Giacoppo S. Diomede F. Ballerini P. Paolantonio M. Marchisio M. (2016a). The secretome of periodontal ligament stem cells from MS patients protects against EAE. *Sci. Rep.* 6:38743. 10.1038/srep38743 27924938 PMC5141419

[B34] Rajan T. S. Giacoppo S. Trubiani O. Diomede F. Piattelli A. Bramanti P. (2016b). Conditioned medium of periodontal ligament mesenchymal stem cells exert anti-inflammatory effects in lipopolysaccharide-activated mouse motoneurons. *Exp. Cell Res.* 349 152–161. 10.1016/j.yexcr.2016.10.008 27737733

[B35] Rajan T. S. Scionti D. Diomede F. Grassi G. Pollastro F. Piattelli A. (2017a). Gingival stromal cells as an in vitro model: cannabidiol modulates genes linked with amyotrophic lateral sclerosis. *J. Cell Biochem.* 118 819–828. 10.1002/jcb.25757 27714895

[B36] Rajan T. S. Scionti D. Diomede F. Piattelli A. Bramanti P. Mazzon E. (2017b). Prolonged expansion induces spontaneous neural progenitor differentiation from human gingiva-derived mesenchymal stem cells. *Cell Reprogram* 19 389–401. 10.1089/cell.2017.0012 29058474

[B37] Rangappa S. Fen C. Lee E. H. Bongso A. Wei E. S. K. (2004). Transformation of adult mesenchymal stem cells isolated from the fatty tissue into cardiomyocytes. *Ann. Thoracic Surg.* 75 775–779. 10.1016/j.matbio.2004.12.004 12645692

[B38] Santos F. Dean W. (2004). Epigenetic reprogramming during early development in mammals. *Reproduction* 127 643–651. 10.1530/rep.1.00221 15175501

[B39] Sargent C. Y. Berguig G. Y. Mcdevitt T. C. (2009). Cardiomyogenic differentiation of embryoid bodies is promoted by rotary orbital suspension culture. *Tissue Eng. Part A* 15 331–342. 10.1089/ten.tea.2008.0145 19193130

[B40] Sathananthan A. H. (2011). Neural stem cells in neurospheres, embryoid bodies, and central nervous system of human embryos. *Microsc. Microanal.* 17 520–527. 10.1017/S1431927611000584 21771387

[B41] Scholl F. A. Dumesic P. A. Barragan D. I. Harada K. Bissonauth V. Charron J. (2007). Mek1/2 MAPK kinases are essential for Mammalian development, homeostasis, and Raf-induced hyperplasia. *Dev. Cell* 12 615–629. 10.1016/j.devcel.2007.03.009 17419998

[B42] Selvaraj V. Plane J. M. Williams A. J. Deng W. (2010). Switching cell fate: the remarkable rise of induced pluripotent stem cells and lineage reprogramming technologies. *Trends Biotechnol.* 28 214–223. 10.1016/j.tibtech.2010.01.002 20149468 PMC2843790

[B43] Slavkin H. C. (1984). Morphogenesis of a complex organ: vertebrate palate development. *Curr. Top. Dev. Biol.* 19 1–16. 10.1016/S0070-2153(08)60392-0 6389024

[B44] Sonoyama W. Liu Y. Yamaza T. Tuan R. S. Wang S. Shi S. (2008). Characterization of the apical papilla and its residing stem cells from human immature permanent teeth: a pilot study. *J. Endodont.* 34 166–171. 10.1016/j.joen.2007.11.021 18215674 PMC2714367

[B45] Taru Sharma G. Dubey P. K. Verma O. P. Pratheesh M. D. Nath A. Sai Kumar G. (2012). Collagen-IV supported embryoid bodies formation and differentiation from buffalo (*Bubalus bubalis*) embryonic stem cells. *Biochem. Biophys. Res. Commun.* 424 378–384. 10.1016/j.bbrc.2012.06.076 22749767

[B46] Thesleff I. Hurmerinta K. (1981). Tissue interactions in tooth development. *Differentiation* 18 75–88. 10.1111/j.1432-0436.1981.tb01107.x 7011890

[B47] Trubiani O. Ciancarelli M. Rapino M. Di Primio R. (1996). Dimethyl sulfoxide induces programmed cell death and reversible G1 arrest in the cell cycle of human lymphoid pre-T cell line. *Immunol. Lett.* 50 51–57. 10.1016/0165-2478(96)02518-7 8793559

[B48] Trubiani O. Guarnieri S. Diomede F. Mariggio M. A. Merciaro I. Morabito C. (2016). Nuclear translocation of PKC alpha isoenzyme is involved in neurogenic commitment of human neural crest-derived periodontal ligament stem cells. *Cell. Signal.* 28 1631–1641. 10.1016/j.cellsig.2016.07.012 27478064

[B49] Xu W. R. Zhang X. R. Qian H. Zhu W. Sun X. C. Hu J. (2004). Mesenchymal stem cells from adult human bone marrow differentiate into a cardiomyocyte phenotype in vitro. *Exp. Biol. Med.* 229 623–631. 10.1177/153537020422900706 15229356

[B50] Yan Q. Gong L. Deng M. Zhang L. Sun S. Liu J. (2010). Sumoylation activates the transcriptional activity of Pax-6, an important transcription factor for eye and brain development. *Proc. Natl. Acad. Sci. U.S.A.* 107 21034–21039. 10.1073/pnas.1007866107 21084637 PMC3000302

[B51] Yasugi S. (1993). Role of epithelial-mesenchymal interactions in differentiation of epithelium of vertebrate digestive organs. *Dev. Growth Diff.* 35 1–9. 10.1111/j.1440-169X.1993.00001.x 37282301

[B52] Yu Y. Casaccia P. Lu Q. R. (2010). Shaping the oligodendrocyte identity by epigenetic control. *Epigenetics* 5 124–128. 10.4161/epi.5.2.11160 20160514 PMC2962897

[B53] Yun J. Song S. H. Park J. Kim H. P. Yoon Y. K. Lee K. H. (2012). Gene silencing of EREG mediated by DNA methylation and histone modification in human gastric cancers. *Lab. Invest.* 92 1033–1044. 10.1038/labinvest.2012.61a 22508389

